# Autoimmune Hemolytic Anemia in Inflammatory Bowel Disease—Report of a Case and Review of the Literature

**DOI:** 10.3390/life12111784

**Published:** 2022-11-04

**Authors:** Aleksandar Toplicanin, Ljubisa Toncev, Vera Matovic Zaric, Aleksandra Sokic Milutinovic

**Affiliations:** 1 Clinic of Gastroenterology and Hepatology, University Clinical Center of Serbia, 11000 Belgrade, Serbia; 2 Faculty of Medicine, University of Belgrade, 11000 Belgrade, Serbia

**Keywords:** inflammatory bowel disease, Crohn’s disease, ulcerative colitis, autoimmune hemolytic anemia, extraintestinal manifestations

## Abstract

A wide spectrum of extraintestinal manifestations (EIMs) can burden patients with inflammatory bowel disease (IBD). EIMs contribute fairly to morbidity and mortality rates in IBD patients. Moreover, EIMs in IBD patients are so frequent that some suggest that IBD should be approached as a systemic disorder. Anemia is very common in IBD patients. The two most common types of anemia in IBD, iron deficiency anemia and anemia of chronic disease, are extraintestinal complications. Autoimmune hemolytic anemia (AIHA) is a rare extraintestinal manifestation of IBD, more frequent in ulcerative colitis (UC) than in Crohn’s disease (CD). In this case-based review of the literature, we present a 36-year-old female patient diagnosed with Crohn’s disease (CD) and Coombs positive AIHA, complicated by pulmonary thromboembolism and successfully treated with anti-tumor necrosis factor (anti-TNF) therapy. The underlying pathophysiological mechanism of AIHA in IBD is unclear. Treatment options for AIHA in IBD patients before biologic therapy included corticosteroids alone or in combination with azathioprine (AZA), methotrexate, and surgical treatment (colectomy and/or splenectomy). Currently, biologic therapy is a promising therapeutic option, especially in corticosteroid refractory or corticosteroid-dependent IBD patients with AIHA.

## 1. Introduction

Inflammatory bowel diseases (IBD), namely Crohn’s disease (CD) and ulcerative colitis (UC), are chronic, relapsing inflammatory diseases of unknown etiology and partially understood pathogenesis. Clinical presentation of IBD in a subset of patients includes a wide spectrum of extraintestinal manifestations (EIMs) that can affect any organ of the human body. Although there are different definitions of EIMs, Vavricka et al. suggest that an EIM is an inflammatory entity caused by the same processes that induce and drive gut inflammation located outside of the intestine in a patient with IBD [1]. A plausible hypothesis on EIM pathogenesis includes immune response triggered by diseased gastrointestinal (GI) mucosa at different sites through shared epitopes of extraintestinal tissues and bowel content including bacteria [1]. The prevalence of IBD-related EIMs varies in different studies, from 6–47% [1,2,3]. The presence of EIMs contributes to both morbidity and mortality in IBD patients [4]. Some authors [1] even suggest that IBD should be considered a systemic disorder, rather than a strictly gastroenterological disease [4]. Most commonly, EIMs affect the musculoskeletal system, eyes, skin, and hepatobiliary tract. Nevertheless, EIMs can affect almost any organ of the human body [1]. EIMs usually occur after IBD is diagnosed, but in up to 24% of patients EIMs can precede the diagnosis of IBD [5]. Extraintestinal complications, on the other hand, should be differentiated from EIMs, as they are not related to the ongoing autoimmune process in IBD patients. Namely, extraintestinal complications arise from the disease itself, as a direct or indirect consequence of intestinal inflammation (e.g., osteoporosis, kidney stones, gallstones, micronutrient deficiencies, peripheral neuropathies) and IBD drug-related side effects.

Anemia is very common in IBD patients. The reported prevalence of anemia in IBD patients varies from 6% to 74% [6,7,8,9], depending on the definition of anemia and on the population considered (i.e., hospitalized vs. outpatients, CD vs. UC, active disease vs. remission). In a systematic review evaluating the prevalence of anemia in IBD patients, the mean prevalence was 17%, but anemia was diagnosed in 16% of outpatients and 68% of hospitalized patients [8]. Data from a more recent systematic review of studies conducted in Europe by Filmann et al., mainly in tertiary centers, revealed the overall prevalence of anemia was 27% in CD and 21% in UC patients. Iron deficiency was present in 57% of the anemic IBD patients [6]. This wide range of anemia prevalence depends on the study design. Namely, anemia is more common in active disease, in females of reproductive age, in CD patients, and in patients from referral centers as a consequence of more severe forms of the disease being treated in specialized IBD centers. The majority of patients experience two main IBD-related types of anemia, iron deficiency anemia (IDA) and anemia of chronic disease (ACD). IDA occurs as a consequence of blood loss and impaired iron absorption, while ACD is a result of the inflammatory process itself. Other less common types of IBD-related anemia include, among others, vitamin B12 and folate deficiency and toxic effects of medications. All of the above-mentioned types of anemia should be considered extraintestinal complications, since they arise as a consequence of an inflammatory process in the bowel, or the medications prescribed to the patient. On the other hand, autoimmune hemolytic anemia (AIHA) is an EIM, since it does not result from IBD inflammation but from the involvement of an organ outside of GI tract. AIHA is rare in IBD patients, and the majority of previously reported cases are diagnosed in patients with UC [10,11,12].

In this case-based review article we report a case of a female patient with colonic CD, Coombs positive AIHA, and pulmonary thromboembolism (PTE), and provide an extensive review of the literature on AIHA in IBD patients together with possible underlying mechanisms and therapeutic strategies used so far.

## 2. Case Report

**Figure 1:**
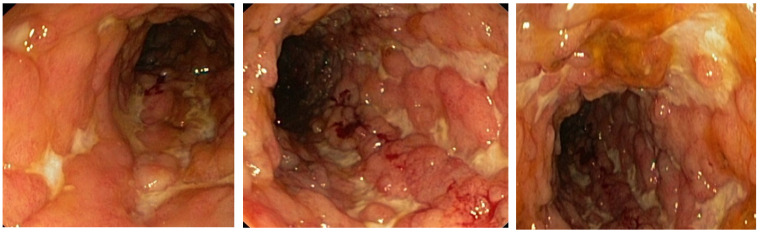
Colon mucosa appearance on initial colonoscopy performed in December 2016. Note deep linear ulcers and lack of healthy mucosa.

In August 2016, a 36-year-old woman presented to the emergency room (ER) of a regional hospital with a 10-day history of diarrhea (up to 5 bowel movements/24 h with occasional hematochezia), abdominal pain, fever (up to 38 C), weakness, chills, vomiting, and altered state of consciousness. During the previous 3 months, she intentionally lost 15 kg. Namely, the patient weight was 88 kg with a body mass index (BMI) of 29.1 when she started the nutritionist-prescribed diet, and afterwards her BMI was 24.1 and her weight reduced to 73 kg. Three weeks before admission, a self-limiting episode of high fever and diarrhea occurred. The patient personal history was negative for chronic diseases, operations, and allergies to medication. The patient denied intake of herbal or non-prescribed medication. Initial vitals gathered in the ER were as follows: blood pressure 90/60 mmHg, pulse 110 beats/minute. Physical examination showed tenderness of both lower quadrants of the abdomen, and chest auscultation identified decreased sounds in the lower right lung field. Laboratory investigations performed on the day of admission showed low hemoglobin (Hgb 35 g/L), mean cell volume (MCV 97 fL), mean corpuscular hemoglobin concentration (MCHC 252 g/L), significant reticulocytosis (15.8%), leukocytosis (45.5 × 10^9^/mL), and thrombocytosis (538 × 10^9^/mL), together with low serum protein (49 g/L), low albumin (18 g/L), and low potassium (2.8 mmol/L), increased lactate dehydrogenase (LDH 1218 U/L), low haptoglobin (0.07 g/L), total bilirubin of 43 umol/L, and direct bilirubin of 16 umol/L. Ferritin was increased to 1130 mcg/mL, while vitamin B12 (147 ng/L) was low and the folate (3.3 mcg/L) level was within normal range. Chest X-ray at admission revealed bronchopneumonia of the lower right lobe. Gallstones were diagnosed on the abdominal ultrasound. Multislice computed tomography (MSCT) scan of abdomen and pelvis revealed slightly enlarged liver and confirmed gallstones. Further laboratory workup revealed positive direct Coombs test (IgG-type warm antibodies and negative anti-C3d), elevated total IgG (21.5 g), positive anti-smooth muscle (ASMA), and positive antinuclear (ANA) autoantibodies in low titers. All other tests including anti-double-stranded deoxyribonucleic acid antibodies (anti-dsDNA), anti-mitochondrial antibodies (AMA), antineutrophil cytoplasmic antibodies (ANCA), antiparietal cell antibodies (APCA), anti-liver kidney microsomal type 1 antibodies (anti-LKM 1), anti-extractable nuclear antigen antibodies (anti-ENA screen), and C3 and C4 complements were within normal range. Venereal disease research laboratory test (VDRL) and Wright test were negative. Screening for human immunodeficiency virus (HIV), hepatitis B virus (HBV), and hepatitis C virus (HCV) was negative. A complete stool infectious panel was negative. A peripheral blood smear showed a low count of erythrocytes with anisocytosis, macrocytosis, and hypochromia. A myelogram confirmed reactive bone marrow, while bone marrow biopsy revealed erythroid hyperplasia. Diagnosis of Coombs positive AIHA was established. The patient was started on corticosteroids (pulse doses of intravenous methylprednisolone 1000 mg/day for first 3 days with subsequent dose tapering), intravenous immune globulin (IVIG), and transfusions of filtered erythrocytes. Antibiotic therapy was initiated for bronchopneumonia (piperacillin-tazobactam) and diarrhea (metronidazole and ciprofloxacin) since it was considered acute enterocolitis. Three weeks after admission a rise in D dimer values (from 4.91 mg/L to 13.45 mg/L) occurred, and MSCT pulmoangiography revealed bilateral segmental PTE with no signs of consolidation or infiltrations in the pulmonary parenchyma, and therapy with low-molecular-weight heparin (LMWH) was initiated. Patient successfully recovered and was discharged in September 2016 with scheduled hematologist consults. In December 2016 she was admitted to the ICU in the University Clinical center of Serbia with a 10-day history of bloody diarrhea (up to 10 stools a day, blood in more than half of the stools), abdominal pain, fever (up to 39 °C), and fatigue. At admission to ER, the patient had fever, shortness of breath, and initial vitals again revealed tachycardia and low blood pressure. Initial laboratory findings revealed normocytic anemia (Hgb 88 g/L, MCV 89 fL) and increased inflammatory parameters (CRP 188 mg/L, ESR 150 mm/h) together with low potassium and serum albumin. There were no signs of hemolysis this time. Upon admission, flexible sigmoidoscopy was performed under clinical suspicion of IBD and revealed deep fibrin-covered ulcerations with pseudopolyps and hyperemic, fragile mucosa. Biopsies were taken and pathological findings confirmed the presence of active chronic ulcerous inflammation. To our surprise, the pathologist suggested CD to be the likely diagnosis. Parenteral corticosteroid therapy was initiated, and the patient was transferred from the ICU to the gastroenterology ward. The patient responded well to the initial corticosteroid treatment, with reduced number of bowel movements. She had 2–3 stools with no blood, no abdominal pain, and no fever. Laboratory findings improved (CRP 23.4 mg/L). Esophagogastroduodenoscopy was performed and showed no endoscopic or pathohistological signs of CD in the upper part of gastrointestinal tract. Since the patient was stable, a total colonoscopy with terminal ileoscopy was performed to assess disease localization, revealing inflamed mucosa of the whole colon with deep, fibrin-covered ulcerations and pseudopolyps. There were no endoscopic signs of CD in the terminal ileum (Figure 1). The histology report was conclusive and confirmed colonic CD.

**Figure 2:**
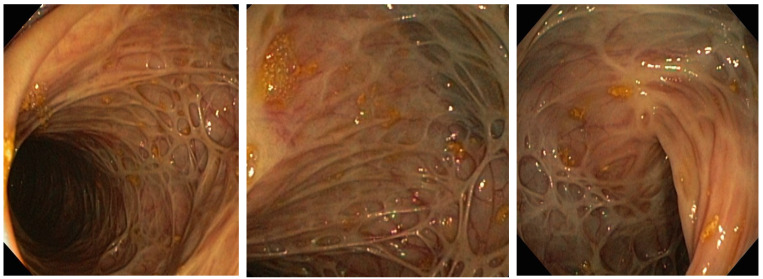
Colon mucosa appearance on colonoscopy performed in December 2018. Note the absence of active Crohn’s disease.

During steroid tapering, at the dose of 35 mg prednisolone, patient had increased number of bowel movements to four–five liquid stools daily followed by increase in CRP levels (141 mg/L) and hemolytic crisis. After detailed workup from an immunologist and hematologist, AIHA was considered as an EIM of CD. In addition, the previous PTE was now considered provoked, since tests for inherited and acquired thrombophilia were negative. Due to steroid-dependent form of disease, we initiated treatment with infliximab in combination with azathioprine. The patient responded to first infliximab infusion, CRP levels decreased (23 mg/L), and she had two formed stools. The patient was discharged and the second induction dose administered after 2 weeks was uneventful. At the third induction dose, on week 6, CD was in clinical remission, with two formed stools daily, no fever, and no abdominal pain, but laboratory signs of another hemolytic crisis were observed with severe anemia (Hgb 60 g/L). We decided to increase the corticosteroid dose to 40 mg prednisolone and blood was taken to determine infliximab trough level and antibodies. Infliximab trough levels were low (0.73 mg/mL) with negative antibodies, so we optimized the treatment regimen by shortening the interval to 4 weeks. The patient responded well, steroids were weaned, and the patient was in clinical and laboratory remission from February 2017 until July 2019. A control colonoscopy in December 2018 confirmed endoscopic remission and low histological activity, as seen in Figure 2. In July 2019, the patient experienced a relapse of CD with abdominal pain and up to 10 liquid bloody stools daily. Infliximab trough levels were low (0.5 mcg/mL) with elevated antibodies (46 IU/mL). The patient was admitted to the Clinic for gastroenterology and hepatology, University Clinical center of Serbia, and started on steroids. The biologic drug was changed within class to adalimumab, as the patient was stable on the first anti-TNF for more than 2 years. Unfortunately, this resulted in a merely partial clinical response with no clinical remission. In October 2019, a colonoscopy revealed severely active disease from the rectum to hepatic flexure, and the biological drug was changed from one of the anti-TNF class to vedolizumab. The patient has since been in clinical, laboratory and endoscopic remission with no more flairs of AIHA. The colonoscopy performed in November 2020 showed endoscopic remission of the disease. Pathohistology confirmed remission with only focal inflammation of low activity. The patient got pregnant, and the pregnancy was uneventful. In July 2021, an elective Caesarian section was performed, and she delivered a male baby (birth weight 4400 g, APGAR score 9). Vedolizumab therapy is ongoing, and the patient is still in remission.

## 3. Discussion

The first report of AIHA in IBD patients was published in 1955 by Lorber et al., who reported four cases of female patients with ulcerative colitis and AIHA [13]. One patient was successfully treated with corticosteroids, while three patients were treated surgically (subtotal colectomy in two cases and subtotal colectomy and splenectomy in one case) and remained disease-free after surgery. All patients were diagnosed with AIHA during relapse of UC. The authors suggested that the possible underlying mechanism of AIHA in these patients included cross-reactivity between antibodies formed against substances (amino acids, carbohydrates, and lipid molecules) absorbed by the diseased colon and antigens on the surface of erythrocytes [13].

### 3.1. Prevalence of AIHA in IBD

The prevalence of AIHA in UC was repeatedly assessed in different studies, and available data suggested that AIHA is diagnosed in 0.2–1.7% of UC patients [3,14,15,16]. In opposition to this, according to the available literature, AIHA seemed extremely rare in CD patients. Namely, until 2020 only seven well-documented reports on AIHA in CD patients were published [17,18,19,20,21,22,23], as seen in Table 1.

In 2020, an abstract on AIHA in IBD was published by Sunkesula and Kundrapa suggesting a higher prevalence of AIHA in IBD patients [24]. They performed a retrospective population-based study using a web platform that includes over 65 million patient data. They identified AIHA in UC, CD, and patients with no IBD. The overall prevalence of AIHA according to their results was 17 per 100,000 patients. A significantly higher prevalence of AIHA was found in IBD patients (125 per 100,000) than in non-IBD patients (18 per 100,000). A total of 350 IBD patients (230 UC and 120 CD) with AIHA were identified.

Based on these results, one could speculate that AIHA is underdiagnosed in both UC and CD patients.

The prevalence of AIHA in UC is not, according to this study, significantly higher compared to CD (131 vs. 115 per 100,000, respectively). The main pitfall of this study is that in IBD AIHA can be also drug-induced or secondary to infection and lymphoproliferative disease [14], as previously reported. Drug-induced AIHA was reported during sulfasalazine, infliximab, and vedolizumab therapy [25,26,27,28,29,30]. The authors of the abstract [24] did not provide further data on the possible underlying cause of AIHA in these patients, which is probably related to their study design.

One of the largest cohorts of UC patients analyzed is in the study by Gumaste et al. [15]. They identified 8 patients with AIHA in 1150 hospitalized UC patients (0.7%). In a Hungarian 25-year follow-up study on 873 patients, 4 UC patients (2 males, 2 females) and no CD patients developed AIHA [3]. Important data were published in 2017 by Uzzan et al. [14]. They performed a multicentric retrospective study aiming to describe the characteristics and outcomes of patients affected by autoimmune cytopenias and IBD. They identified 14 IBD-related AIHA cases (13 in UC and 1 in CD). The prevalence of AIHA in UC patients was as high as 150 per 100,000 patients. This prevalence is higher than the reported lifetime prevalence in the general population (17/100,000 for AIHA in a Danish population-based cohort study) [31]. Other reports on AIHA in UC include case reports and smaller case series [32,33]. Since available data on AIHA in CD patients consists of case reports, no estimates on prevalence could be given.

### 3.2. Sex-Based Differences in IBD Patients with AIHA

In a recent review by Rustgi et al. that focused on sex-based differences in IBD, the authors comprehensively reviewed different aspects of the topic. The authors provided evidence on sex-based differences in pathogenesis, disease course, EIM epidemiology, and response to therapy that have been recognized [34]. Data on sex-based differences in AIHA occurrence in IBD patients is contradictory. In the first case series of UC and AIHA by Lorber et al., all patients were female [13]. Other published studies in UC and AIH patients provided conflicting data. In a study by Gumaste, there was clear female predominance, as seven out of eight patients were female [15]. Contrary to this result, in the French study, only 5 out of 14 (35.7%) patients with IBD and AIHA were female [14]; that is consistent with the results published by Bardana et al. where 5 out of 7 patients were male [35]. In CD patients with AIHA, there is a clear male predominance, considering that, out of seven previously described patients, six were males and one was female. Our patient was also female. Due to the limited number of cases published so far, no definite conclusion can be drawn. It could be that in females of reproductive age, anemia is common and AIHA is underdiagnosed because screening is not conducted, but we could not exclude other possible unknown reasons for male predominance [17,18,19,20,21,22,23].

### 3.3. Age at Onset of AIHA

Age at onset of UC in a study by Gumaste et al. ranged from 11 to 50 years (mean 25 years), and, at diagnosis of anemia, from 23 to 53 (mean 35 years) [15]; that did not differ from results by Uzzani et al., where age at onset of AIHA was 23.4–56.9 (mean 31.2 years). In a study by Bardana et al., age range at onset of AIHA was 11–70 (mean 37 years) [35]. Therefore, it can be concluded that age does not seem to play a crucial role in the onset of AIHA.

### 3.4. Onset of AIHA in IBD Patients

AIHA may precede, accompany, or follow the onset of UC [36]. It may even occur several years after colectomy [37]. In the first publication from Lorber, AIHA followed the onset of UC in all four patients, with different mean duration of colitis before AIHA occurrence [13]. According to the study by Gumaste [15], the mean duration of colitis prior to diagnosis of AIHA was 10 years, while in the study by Uzzan, the IBD preceded diagnosis of AIHA in 10 cases, 3 patients had a concomitant diagnosis, and AIHA was diagnosed prior to IBD in 1 patient [14]. The study by Bardana et al. identified 18 patients with UC that were subsequently diagnosed with AIHA [35].

In CD patients, AIHA preceded CD in three cases, the concomitant diagnosis was established in one patient, and the diagnosis of CD preceded the AIHA diagnosis in three cases. Thus, in CD patients, AIHA can precede, accompany, or follow the diagnosis of CD. In our patient, the diagnosis should have been concomitant, but instead the AIHA diagnosis preceded the diagnosis of IBD [17,18,19,20,21,22,23].

### 3.5. Disease Localization in IBD Patients with AIHA

The majority of UC patients with AIHA had pancolitis, as confirmed by different studies [13,32,33]. In a study from Mount Sinai Hospital, five of eight patients had pancolitis at the time AIHA was diagnosed, two had left-sided colitis (one progressed to pancolitis), and a single patient had only proctosigmoiditis [15]. In a study by Uzzan et al., 10 out of 13 UC patients had pancolitis, and the other 3 had left-sided colitis [14]. In a study by Lakatos, there are no data on disease localization, but this study focused on overall incidence of EIM and not specifically on AIHA [3].

In CD patients with AIHA, four patients had ileocolitis [18,20,21,22], one had colitis [17], while two patients had disease localized in the ileum [19,23]. In the French cohort, a single CD patient had ileocolitis [14]. Since the colon was affected in the majority (six out of seven) of CD patients with AIHA, and keeping in mind higher reported incidence of AIHA in UC, one could speculate that colonic inflammation plays a role in the occurrence of AIHA in IBD patients.

### 3.6. IBD Activity and Occurrence of AIHA

In the first case series of UC and AIHA by Lorber et al., all patients had active IBD at the time AIHA was diagnosed [13]; that was supported by the findings of others [16]. Uzzan et al. reported that AIHA activity followed the IBD activity in 71% of cases [14]. Nevertheless, this study dealt with autoimmune cytopenia in IBD, thus in three patients AIHA was proven to be secondary to low-grade lymphoid malignancy, and in two patients resulted from CMV primo-infection [14]. All patients with CD had active disease at the time of AIHA diagnosis [17,18,19,20,21,22,23]. All of the above-mentioned data suggest that AIHA is a true EIM in IBD patients

### 3.7. Other EIMs in AIHA Patients with IBD

In a study by Gumste, three female patients had other EIMs (erythema nodosum, pyoderma gangrenosum, and arthritis) together with AIHA [15], while in a study by Uzzan three patients had other EIMs, but the authors did not specify which [14]. In a study by Bardana and Piotrofsky, EIMs are not listed separately, but the presence of erythema nodosum, arthritis, and arthralgia is mentioned in at least 3 out of 18 patients [35].

Surprisingly, despite that primary sclerosing cholangitis (PSC) is a rare EIM in CD, it was associated with AIHA in two out of seven patients, while serology was positive for pANCA antibodies in three patients. Namely, recent meta-analysis results show that pooled prevalence of PSC in patients with UC is higher than in those with CD (2.47% vs. 0.96%, respectively), and remains highest in IBD-unclassified patients (5.01%) [36]. In patients with UC and AIHA, our search of the literature identified a single case report by Naqvi et al. of an 18-year-old man diagnosed with ulcerative colitis, AIHA, and PSC during the same hospitalization [11].

### 3.8. Treatment of AIHA and IBD

In a study by Uzzan, conservative therapy of IBD-related AIHA comprised of transfusion in 9 cases; steroids in 13; IVIG was administered in 2 patients; AZA was administered in 3; and 2 patients had splenectomy. Other treatments used were plasma exchange in one patient, infliximab also in one, sirolimus in one, and cyclophosphamide in one [14].

In a study by Ramakrishna published in 1994, all previous cases of UC and AIHA were summarized, and therapeutic strategies included corticosteroids in 18, combination of corticosteroids and azathioprine in 16, corticosteroids and danazol in 1, splenectomy in 8, combined splenectomy and colectomy in 6, and colectomy in 10 patients [32].

Gumaste et al. reported favorable outcome in seven out of eight patients [15]. One patient responded to steroids, two had a splenectomy, and in four both colectomy and splenectomy were performed.

An interesting observation is that of Uzzan et al. [14] concerning two UC patients who underwent a subtotal colectomy with ileorectal anastomosis, and subsequently presented active AIHA, along with refractory proctitis. In both of these cases, secondary proctectomy induced remission of AIHA. Radical surgery led to a complete remission in some patients with AIHA. The efficacy of cyclosporine and anti-TNF agents was also reported.

Giannadaki et al. reported that out of five AIHA patients with IBD, three were treated successfully with corticosteroids, one patient responded to corticosteroids with azathioprine, and in one case a total colectomy, together with a splenectomy, led to a remission of AIHA and UC [16].

Remission of AIHA and CD was conceded with corticosteroid therapy in three cases, corticosteroids and azathioprine in one case, methotrexate alone in one case, and the remaining two patients were treated surgically [17,18,19,20,21,22,23]. In one case reported by Plikat et al. [20], anti-TNF therapy failed to induce a remission of AIHA and CD, and the patient was treated surgically because low-grade dysplasia was detected in random colonic biopsies. There is no detailed information on the dosing regimen of anti-TNF therapy in this patient.

Namely, standard dosing of infliximab is weight-based at 5 mg/kg administered intravenously. The regimen includes an induction phase (doses given at weeks 0, 2, and 6) and maintenance treatment (doses given every 8 weeks). Large interindividual variability in the pharmacokinetics of infliximab were previously reported together with well-defined factors associated with high drug clearance, such as degree of systemic inflammation, low serum albumin, and high body weight [37]. The concomitant use of immunosuppressants reduces infliximab clearance. A recent review summarized available data on infliximab pharmacokinetics and pharmacodynamics, and the authors reported that a positive relationship was noted between the serum infliximab concentration and clinical effect in both adult and pediatric IBD patients [37]. Early trough level determination in week 6 and subsequent optimization of therapy by shortening the interval, which aims to increase drug concentration, most likely led to therapeutic success with our patient.

Our patient was treated successfully using anti-TNF in the first line. A loss of response to infliximab occurred after 29 months of therapy and was attributable to anti-infliximab antibody formation. After that we introduced adalimumab as a second anti-TNF drug, but the patient was not responding. The patient was a secondary non-responder to anti-TNF, thus vedolizumab was introduced. Vedolizumab led to clinical, laboratory, and endoscopic remission of both CD and AIHA.

Before biologic therapy was introduced, the majority of AIHA patients with IBD underwent surgery in order to achieve stable disease remission, while a limited number were successfully treated with corticosteroid therapy. Biologic therapy, although data on the subject are scarce, seems like a promising therapeutic option in these patients.

### 3.9. Possible Underlying Mechanism of AIHA in IBD

The mechanism linking ulcerative colitis and AIHA is not fully understood. One of the current hypotheses suggests that the damaged colon wall in UC absorbs nonspecific, non-red-cell antigens that then induce an immunological response and further cross-reactivity of antibodies produced against those antigens and red blood cells in circulation [32,33,38,39,40]. Other possible mechanisms include the damage of erythrocytes in the GI tract, with subsequent absorption of altered erythrocyte antigens through the diseased colonic wall, resulting in a hemolytic process [38]. Cross-reactivity between autoantibodies produced against diseased colonic mucosa, and antigens on the surface of erythrocytes, is another proposed mechanism [36]. UC, as a chronic inflammatory disease, leads to alteration in the whole immune system, which might lead to the appearance of a clone of immunocompetent cells that could produce antibodies directed at antigens on the surface of erythrocytes [38]. In some patients, there were reports of AIHA even years after proctocolectomy was conducted, because of UC. This indicates that in UC patients there might be an underlying predisposition to an altered immunological response which can result in more than one autoimmune disease [32,41].

Pathogenesis of AIHA as an EIM of CD is not fully understood. Eliam et al., in the first case report of AIHA in a CD patient, emphasized the importance of both immunological and genetic mechanisms in the pathophysiology of these two co-existing conditions. They reported an increased frequency of HLA-B8, DR3, and DRw52a alleles in patients with PSC, and their patient had the same allele combination [17]. One of the other hypotheses outlines the importance of colon involvement. According to this hypothesis, there might be cross-reactivity of autoantibodies produced as a response to antigens that invade the wall of the colon, causing autoimmune inflammation and the antigens on the surface of erythrocytes [18,19,20]. An important thing to note is that AIHA as an EIM of CD is associated with p-ANCA positivity and PSC in some cases, suggesting similar biological activity of CD in those patients to ulcerative colitis [17,18,19]. Taking all these facts and the facts from our case into account, we can conclude that the key to controlling AIHA is controlling the inflammation in the colon.

### 3.10. IBD, AIHA, and Thromboembolic Events—Just a Coincidence or Is There More to It?

IBD is considered a significant risk factor for venous thromboembolism (VTE). A meta-analysis of ten cohort studies and one case–control study revealed a 2.2-fold increased risk of VTE in IBD patients, with no statistically significant difference found in the risk for VTE between UC and CD patients. The most common types of VTE among IBD patients are deep venous thrombosis (DVT) and PTE, but there were reports of some uncommon sites such as retinal, hepatic, mesenteric, and cerebral veins [40]. The pathophysiological mechanism is probably multifactorial, but not completely elucidated. Local and systemic inflammation in IBD promotes an IL-6-mediated procoagulant state. Increased platelet numbers, together with their abnormal spontaneous aggregation, also contribute to the development of VTE, along with a low degree of fibrinolysis in IBD patients, as is suggested in some studies. Other possible VTE contributing factors include dehydration and the use of corticosteroid therapy [41,42], as well as previously reported, well-known risk factors (i.e., older age, hospitalization, surgery, and IBD flares) [43]. Furthermore, the risk for VTE in IBD patients is significantly higher than in patients with other acute or chronic gastrointestinal diseases, including GI malignancies [41]. Our patient had more than one risk factor for developing PTE. Currently, AIHA is being increasingly recognized as an important risk factor for VTE. Ramagopalan et al. examined the incidence of VTE among patients hospitalized because of immune-mediated diseases and compared them with three control groups hospitalized for other conditions. They found a nearly three-fold increase in risk for VTE among AIHA patients compared to controls. Newer studies emphasize the importance of surveillance and prophylaxis for VTE among AIHA patients, since available guidelines for managing this condition do not provide such recommendations [44].

## 4. Conclusions

AIHA is a very rare, but potentially life-threatening EIM of CD. In the majority of cases of IBD and AIHA, it was related to active IBD, and resolving gut inflammation leads to the resolution of AIHA. Treatment options in the pre-biologic therapy era usually consisted of corticosteroids and surgery, while currently biologic therapy is also an effective option. Monitoring of drug trough levels should be used, and early optimization of the drug dose applied if necessary. It seems that, from the large population-based, although retrospective data, AIHA in CD might be more frequent and underdiagnosed in clinical practice. A proactive search for AIHA and well-designed prospective studies are needed in order to clarify if AIHA is a rare or underdiagnosed EIM in CD patients

## Figures and Tables

**Table 1:** Characteristics of previously reported cases of CD patients with AIHA.

	Eilam, 1993 [17]	Hochman, 2002 [18]	Ng, 2004 [19]	Plikat, 2005 [20]	Tsiopoulos, 2009 [21]	Kallel, 2009 [22]	Park, 2014 [23]
Age, gender	41, male	11, male	44, male	29, male	57, male	21, male	41, female
Coombs test	+	+	+	+	+	+	Negative
Disease that occurred first	AIHA	CD	CD	Concomitant	AIHA	CD	CD
Interval between diagnosis	7 years	2 years	2 years	0	unknown	9 months	4 years
Other diseases or conditions	Primary sclerosing cholangitis	*	*	*	Thrombosis of the ophthalmic artery	Primary sclerosing cholangitis	*
Therapy that did not induce remission	CS	CS mesalamine and 6-MP	Mesalamine	CS, MTX, and cyclosporine anti-TNF	*	Mesalamine	*
Therapy that induced remission	Splenectomy	MTX	CS + AZA	Surgery (subtotal colectomy)	CS	CS	CS
CD localization	Colitis (cecum and ascending colon)	Ileocolitis	Ileitis with perianal abscess and fistula	Ileocolitis with proximal disease (duodenum)	Ileocolitis	Ileocolitis with perianal fistula	Ileitis

CS—corticosteroids; AZA—azathioprine; MTX—methotrexate; MP—6 mercaptopurine, +—positive; *—not reported.
